# INTRODUCING EFRAILTY: SIMPLIFYING SELECTION OF FRAILTY ASSESSMENT TOOLS

**DOI:** 10.1093/geroni/igad104.3749

**Published:** 2023-12-21

**Authors:** Megan Cheslock, Stephanie Sison, Lily Zhong, Natalie Newmeyer, Vaishnavi Raman, Ariela Orkaby, Andrea Schwartz, Dae Kim

**Affiliations:** Edith Nourse Rogers Memorial Veterans Hospital (VA Bedford), Bedford, Massachusetts, United States; UMass Memorial Medical Center - University Campus, Worcester, Massachusetts, United States; University of Connecticut School of Medicine, Farmington, Connecticut, United States; Hinda and Arthur Marcus Institute for Aging Research, Brookline, Massachusetts, United States; University of Toronto, Toronto, Ontario, Canada; VA Boston Healthcare System, Boston, Massachusetts, United States; VA Boston Healthcare System, Boston, Massachusetts, United States; Hinda and Arthur Marcus Institute for Aging Research, Brookline, Massachusetts, United States

## Abstract

Health care providers recognize the importance of frailty assessment for older adults, but they may be unfamiliar with which frailty assessment tool to use. We sought to create an accessible website to assist clinicians in choosing an effective, evidence-based frailty screening tool. We selected commonly used frailty tools based on the literature and worked with a web designer to develop the eFrailty website prototype. A short description of each tool’s key features and estimated time for assessment is included for each frailty tool. An algorithm based on differences in patient characteristics, clinical scenarios, available information, and time for assessment was created to guide users. Modeled after the highly popular ePrognosis website, eFrailty is designed to guide clinicians to select the ideal frailty tool for their clinical context. The site prompts clinicians to choose between patients considering stressful treatment (e.g., major surgery), or patients with or without serious illness. Depending on available information, clinicians choose between ‘Self reports/records only,’ or ‘Performance tests available’ including cognitive screens or physical performance testing. Alternatively, Clinicians may use the eFrailty comparison table which builds on the work of several systematic reviews of frailty identification tools to easily select the best instrument for their patient. A recent addition to the site is a crosswalk to compare scores between different frailty assessment tools. Future directions for eFrailty include beta testing to gather clinician input from point of care use.

